# Synthesis and Characterization of Poly(Lactic-Co-Glycolic Acid)–Paclitaxel (PLGA-PTX) Nanoparticles Evaluated in Ovarian Cancer Models

**DOI:** 10.3390/pharmaceutics17060689

**Published:** 2025-05-23

**Authors:** Sylwia A. Dragulska, Maxier Acosta Santiago, Sabina Swierczek, Linus Chuang, Olga Camacho-Vanegas, Sandra Catalina Camacho, Maria M. Padron-Rhenals, John A. Martignetti, Aneta J. Mieszawska

**Affiliations:** 1 Department of Chemistry and Biochemistry, Brooklyn College, Brooklyn, NY 11210, USA; 2 Rudy L. Ruggles Biomedical Research Institute, Nuvance Health, Danbury, CT 06810, USA; 3 Department of Obstetrics, Gynecology and Reproductive Sciences, Larner College of Medicine, University of Vermont, Burlington, VT 05401, USA; 4 Department of Genetics and Genomic Sciences, Icahn School of Medicine at Mount Sinai, New York, NY 10029, USAmaria.padronrhenals@mssm.edu (M.M.P.-R.)

**Keywords:** poly(lactic-co-glycolic acid), paclitaxel, nanoparticles, ovarian cancer

## Abstract

We developed a novel biodegradable poly(lactic-co-glycolic acid) (PLGA) polymer chemically modified with paclitaxel (PTX) to form a PLGA-PTX hybrid. Pre-modification of PTX enhanced its loading in PLGA-PTX nanoparticles (NPs). **Background/Objectives**: PTX is one of the most effective chemotherapy agents used in cancer therapy. The primary mode of PTX’s action is the hyperstabilization of microtubules leading to cell growth arrest. Although highly potent, the drug is water insoluble and requires the Cremophor EL excipient. The toxic effects of the free drug (e.g., neurotoxicity) as well as its solubilizing agent are well established. Thus, there is strong clinical rationale and need for exploring alternative PTX delivery approaches, retaining biological activity and minimizing systemic effects. **Methods**: The PTX modification method features reacting the C-2′ and C-7 residues with a linker (succinic anhydride) to produce easily accessible carboxyl groups on the PTX for enhanced coupling to the hydroxyl group of PLGA. The PLGA-PTX hybrid, formed via esterification reaction, was used to formulate lipid-coated PLGA-PTX NPs. As proof of concept, the PLGA-PTX NPs were tested in ovarian cancer (OvCA) models, including several patient-derived cell lines (PDCLs), one of which was generated from a platinum-resistant patient. **Results**: The PLGA-PTX NPs critically remained stable in water and serum while enabling slow drug release. Importantly, PLGA-PTX NPs demonstrated biological activity. **Conclusions**: We suggest that this approach offers both a new and effective PTX formulation and a possible path towards the development of a new generation of OvCA treatment.

## 1. Introduction

Ovarian cancer (OvCA) is one of the most common and deadliest cancers in women [1]. It is a heterogeneous disease, as it consists of various subtypes with different molecular and clinical characteristics [2]. The treatment of OvCA typically involves a combination of surgery and chemotherapy. The majority of cases are diagnosed as late-stage disease due to a lack of early symptoms and have a poor 5-year survival rate of ~30%, almost always secondary to the development of treatment resistance [3,4,5]. Common drugs used for OvCA treatment include paclitaxel (PTX) and carboplatin [6,7]. Most tumors initially show a response but then recur [8]. The main shortcomings of therapy include key dose-limiting drug toxicities that often lead to drug resistance and cancer relapse [9,10].

PTX is a potent chemotherapy agent that disrupts cell division, and it is used to treat various cancers, including ovarian, breast, lung, and pancreatic cancer [11,12,13]. It belongs to a class of drugs known as taxanes, which work by interfering with the normal function of microtubules, essential components of the cell’s structure involved in cell division [14]. By stabilizing microtubules, PTX prevents them from disassembling, effectively disrupting the cell division process and causing the cancer cells to remain arrested in the division phase, leading to their death [15]. PTX is not water soluble; therefore, it is typically formulated in a solvent called Cremophor EL, which helps PTX dissolve. However, Cremophor can cause allergic reactions in some patients and has been associated not only with severe anaphylactoid hypersensitivity reactions but also with hyperlipidaemia, abnormal lipoprotein patterns, or aggregation of erythrocytes and peripheral neuropathy [16,17]. Also, resistance to PTX can develop, often due to changes in the microtubules or in the efflux pumps (such as P-glycoprotein) that expel the drug from cancer cells [18]. Therefore, there is a clinical justification and necessity to investigate alternative methods of delivering PTX that preserve its biological activity while reducing systemic side effects.

Nanoparticles (NPs) can potentially minimize systemic toxicity and enhance the therapeutic efficacy of drugs, as well as solubilize hydrophobic agents [19,20,21,22,23]. PTX-encapsulating NP carriers, including liposomes [24,25,26] or polymeric micelles [18,27], protein [28], and RNA-based complexes [29,30], made through physical encapsulation of PTX or chemical conjugation, have been explored [31]. Second-generation NP systems are based on slow-releasing polymers, such as poly(lactic-co-glycolic acid) (PLGA). The excellent physicochemical properties of PLGA and its low toxicity prompted approval of PLGA by the United States Food and Drug Administration (FDA) and the European Medicines Agency, making the polymer an attractive choice for many NP formulations [32]. The PLGA polymer is biodegradable and biocompatible and allows for the controlled and sustained release of encapsulated drugs, resulting in prolonged therapeutic effects of NP formulations that can sharply reduce the frequency of drug administration, and many are at the pre-clinical stage of development [33,34,35,36]. PLGA-based NPs encapsulating PTX have been explored, and the drug loading into the PLGA matrix was based primarily on physical methods [37,38].

Herein, we explore an alternative strategy that relies on forming the PLGA-PTX hybrid first by chemical attachment of PTX to PLGA and then formulating the NPs using the PLGA-PTX hybrid. Importantly, the novel use of a succinic acid linker with PTX provided easily accessible carboxyl groups, which sterically facilitated the coupling reaction to PLGA. This resulted in an increased payload of PTX in the final NP construct. The technology allowed for solubilizing the PTX in a biologically benign polymer, resulting in a complete replacement of a toxic Cremophor EL excipient, and providing a NP system with predictable drug release kinetics. As proof of concept, the NPs were tested in OvCA models, as PTX is the standard care for primary OvCA management.

## 2. Materials and Methods

### 2.1. Materials

Trifluoroacetic acid 99% (TFA) and Dicyclohexylcarbodiimide 99% (DCC) were purchased from Acros Organics (Waltham, MA, USA). Triethylamine, 4-dimethylaminopyridine (DMAP) and succinic anhydride 99% were purchased from Alfa Aesar (Haverhill, MA, USA). Poly(D,L-lactide-co-glycolide) (PLGA) LA: GA (50:50) mol wt. 1000–5000 and 5000–10,000 were purchased from PolySciTech (West Lafayette, IN, USA). Paclitaxel (PTX) was purchased from TSZ Chem (Framingham, MA, USA) > 99.5%, and Slide-A-Lyser MINI dialysis units were purchased from Thermo Scientific (Waltham, MA, USA). DSPC and DSPE-PEG2000 were purchased from Avanti Lipids (Alabaster, AL, USA).

All solvents were bought and used without further purification. Methylene chloride (DCM), N, N-dimethylformamide (DMF), and methanol were purchased from Fischer Scientific (Waltham, MA, USA) (ACS grade). Acetonitrile (ACN) HPLC grade was purchased from Acros Organics (Waltham, MA, USA). Ethyl alcohol 190 proof was purchased from Koptec (King of Prussia, PA, USA).

### 2.2. Instruments

NMR Bruker 400 MHz Ultra Shield instrument (Bruker Corporation, Billerica, MA, USA).
LC/MS: Ultra High-Performance Liquid Chromatography System Agilent Technologies 1200 series Accurate-Mass TOF LC/MS 6220; Agilent QDB-C18 column, 4.6 × 150 mm, 5 µm (Agilent Technologies, Santa Clara, CA, USA).
HPLC: High-Performance Liquid Chromatography FLEXAR System Perkin Elmer (Waltham, MA, USA)
DLS: Dynamic Light Scattering (Brookhaven Instrument Corporation, Holtsville, NY, USA)
TEM: FEI Titan Themis 200 (Molecular Devices, San Jose, CA, USA)
Plate reader: SpectraMax M3 (Molecular Devices, San Jose, CA, USA)
Confocal microscope: LSM 880 Airyscan Fast Live Cell (Carl Zeiss Microscopy GmbH, Jena, Germany)

### 2.3. Synthesis of 2′,7-Disuccinyltaxol

**Figure 1:**
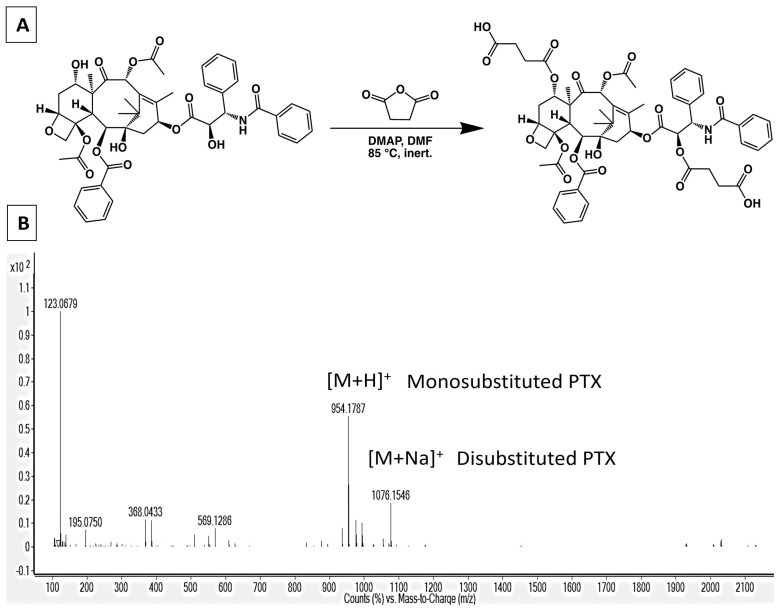
(**A**) Schematic of the synthesis of PTX-succinic acid linker. (**B**) HRMS−ESI: For the mixture of 2′−succinyltaxol and 2′,7−disuccinyltaxol, peaks were found at *m*/*z* 954.1787 [M + H]^+^ and *m*/*z* 1076.15 [M + Na]^+^, respectively.

First, 20 mg (0.023 mmol) of PTX, 28 mg (0.281 mmol) of succinic anhydride, and 2.86 mg (0.023 mmol) of DMAP were dissolved in 3 mL of DMF, and the reaction was carried out for 24 h at 85 °C. After that, the mixture was dried in a rotavapor. The residue was treated with 10 mL of cold nanopure water, stirred for 20 min, and filtered. The precipitate was dissolved in acetone, and the final crystals were collected after adding cold water. The reaction yield was 81%, and the mass of the obtained product was 18 mg of the mixture 2′-succinyltaxol and 2′, 7-disuccinyltaxol (Figure 1A). HRMS-ESI: *m*/*z* [M + H]^+^ calc. for C_51_H_55_NO_17_: 953.3470; found: 954.1787 for 2′-succinyltaxol, and HRMS-ESI: *m*/*z* [M + Na]^+^ calc. for C_55_H_59_NO_20_: 1053.36; found: 1076.15 for 2′, 7-disuccinyltaxol, respectively (Figure 1B).

### 2.4. Synthesis of PTX-PLGA Hybrid with Succinic Acid Linker (Method 1)

**Figure 2:**
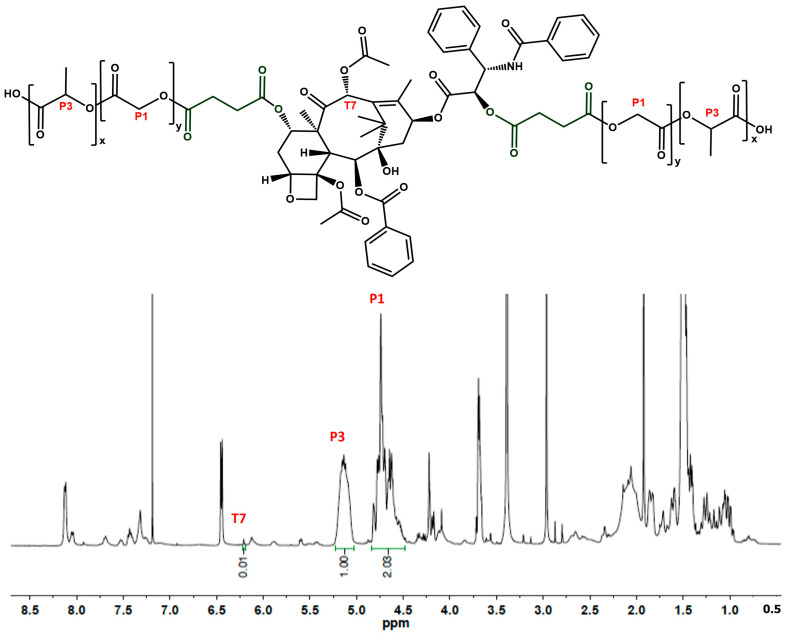
^1^H NMR (400 MHz, CDCl_3_) of the PLGA-PTX hybrid with a succinic acid linker. The quantification was based on the PLGA (7.5 kDa) peak at ~4.9 ppm (integration: 2.03) and the PTX peak at ~6.25 ppm (integration: 0.01).

Initially, 83.3 mg (0.011 mmol) of 5–10 kDa PLGA, 11.7 mg (0.011 mmol) of 2′, 7-disuccinyltaxol, 9.16 mg (0.044 mmol) of DCC, and 5.42 mg (0.044 mmol) of DMAP were dissolved in 5 mL of anhydrous DCM, and the reaction was carried out for 24 h at room temperature. Next, the mixture was dried in a rotavapor, the residue was dissolved in ACN and added dropwise to cold methanol. The precipitate was centrifuged and washed two times with cold methanol. The reaction yield was 77%, and the mass of the obtained product was 73 mg. The ^1^H NMR spectrum used for establishing the PLGA:PTX ratio is presented in Figure 2.

### 2.5. Synthesis of PTX-PLGA Hybrid Without Succinic Acid Linker (Method 2)

**Figure 3:**
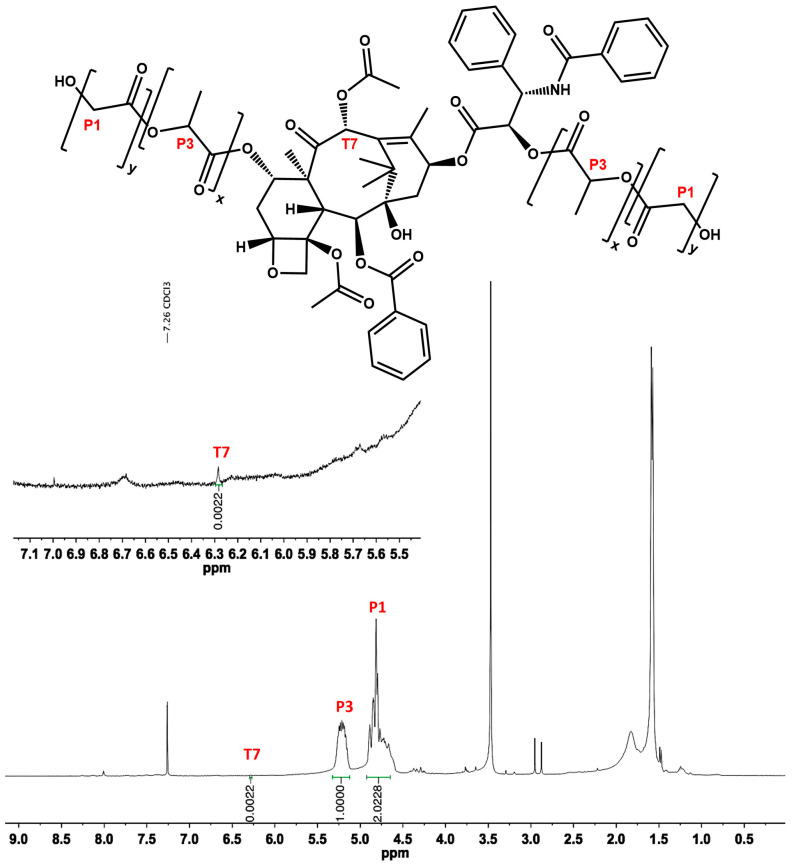
^1^H NMR (400 MHz, CDCl_3_) of the PLGA-PTX hybrid where PLGA is directly bonded to PTX via an ester bond. The quantification was based on the PLGA (2.5 kDa) peak at ~4.9 ppm (integration: 2.022) and the PTX peak at ~6.25 ppm (integration: 0.002).

First, 102 mg (0.04 mmol) of 1–5 kDa PLGA, 0.08 mol of Paclitaxel (PTX), 0.08 mol of DCC, and 0.04 mol of DMAP were dissolved in 1 mL of anhydrous DMF, and the reaction proceeded for 12 h at room temperature. After that, the mixture was filtered to eliminate urea. The filtrate was added dropwise to cold methanol, and it was left at −2 °C overnight to precipitate the PLGA-PTX hybrid. Next, the precipitate was centrifuged and washed three times with cold methanol. The isolated product (65.3 mg) represented a yield of 63.7%. The ^1^H NMR spectrum used for establishing the PLGA:PTX ratio is presented in Figure 3. The calculations were performed according to Equations (1)–(3).

### 2.6. Method to Calculate PLGA:PTX Ratio

The PLGA:PTX ratio was established from NMR studies based on the average PLGA integration peak (Int) and the number of PLGA monomers (PLGA_Mon_) present. These values were used to calculate the integration per PLGA monomer (Int_Mon_). The Int was calculated using the following equation:

$$Int=\left(\frac{{P1}_{Exp}}{{P1}_{Theo}}+P2\right)\times \frac{1}{2}$$

and it involves the normalization of the NMR integration of the -CH- group in PLGA (denoted as P2) to that of ^1^H NMR. This normalization also accounts for any experimental variations associated with the -CH_2_- group in PLGA (P1).

For quantifying the PLGA monomers (PLGA_Mon_), we considered an average molecular weight (M.W.) of the PLGA per monomer (2500 Da or 7500 Da), relative to the M.W. of the PLGA subunit (166 Da):

$${PLGA}_{Mon}=\frac{{M.W.}_{PLGA}}{166}$$

The integration per PLGA monomer was determined from the equation:

$${Int}_{Mon}=\frac{Int}{{PLGA}_{Mon}}$$

The PLGA_Mon_:PTX ratio was calculated from Int_Mon_ and the experimental PTX integration value of singlet T7.

### 2.7. Synthesis of the PLGA-PTX NPs

The PLGA-PTX NPs were synthesized via a nanoprecipitation method [39]. First, 5 mg of PLGA-PTX hybrid was dissolved in 2.5 mL of acetonitrile to achieve the concentration of 2 mg/mL. Next, the PLGA-PTX solution was added dropwise to the mixture of the following lipids: phospholipids (DSPE; 0.02 molar ratio to total lipids) and 1 mg of DSPC-PEG (7:3 molar ratio) in 5 mL of 4% ethanol in water at 60–70 °C. The PLGA-PTX NPs were allowed to stir overnight, and then the NP solution was washed three times with water using Vivaspin centrifugal concentrators (MWCO 100 kDa). The NP sample was condensed to achieve the volume of ~1 mL and characterized by TEM and DLS. The PTX concentration was analyzed using HPLC.

### 2.8. Characterization of Size and Surface Charge of PLGA-PTX NPs

#### 2.8.1. Dynamic Light Scattering (DLS) and Zeta Potential Measurements

The size of PLGA-PTX NPs and zeta potential were analyzed by dynamic light scattering (DLS) equipped with ZetaPals (Brookhaven Instrument Corporation). The concentrated NP solution was diluted in nanopure water to 1.2 mg NPs/mL and analyzed for hydrodynamic diameter and surface charge. The measurements were done in triplicates.

#### 2.8.2. Transmission Electron Microscopy (TEM) Imaging

The core size and morphology of the NPs were analyzed using TEM (FEI Titan Themis 200 kV). To this end, the NP sample was mixed with the acetate buffer (0.125 M CH_3_COONH_4_, 0.6 mM (NH_4_)_2_CO_3,_ and 0.26 mM tetrasodium EDTA at pH 7.4). Next, 10 μL of the NPs’ sample was negatively stained with 10 μL of 2% (*w*/*v*) phosphotungstic acid, and a drop of as-prepared NP solution was cast on a 200-mesh carbon-coated copper grid (Electron Microscopy Sciences, Hatfield, PA, USA) and left to dry. Right before the imaging, the grid was cleaned with Fischione M1070 NanoClean to remove any organic contamination.

### 2.9. Characterization of PTX Loading in PLGA-PTX NPs

**Figure 4:**
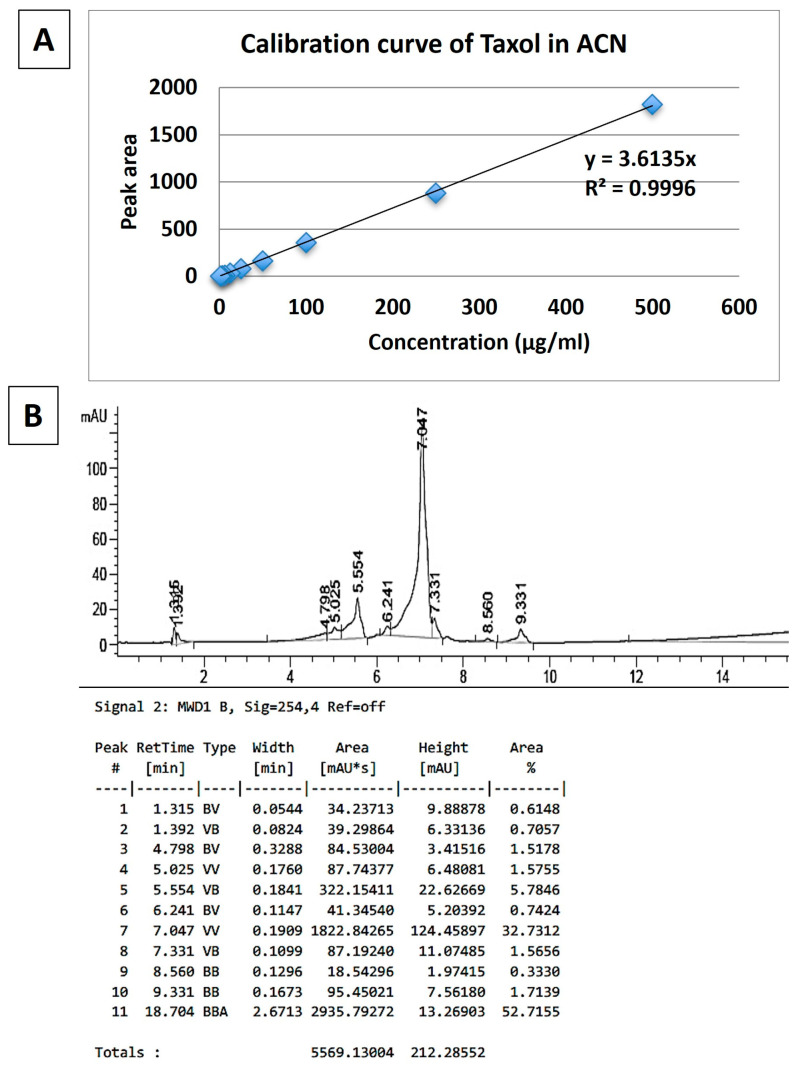
HPLC chromatogram of free PTX (500 ppm) treated with microwaves. (**A**) The calibration curve for free PTX. (**B**) The peak at a retention time of 7.047 min represents the major PTX peak.

HPLC calibration curve for PTX. First, 1 mg of PTX was dissolved in 1 mL of acetonitrile followed by the addition of 1 mL of 1 M NaOH. Then, the solution was microwaved for 15 min at 45 °C. After that, the mixture was neutralized with 10% HCl and lyophilized. PTX was dissolved in 1 mL of acetonitrile and used in series dilutions to prepare solutions at 500, 250, 100, 50, 25, 12.5, 6.25, 3.125, and 1.512 ppm concentrations. The solutions were analyzed using HPLC (FLEXAR System, Perkin Elmer). The mobile phase consisted of acetonitrile:water in gradient elution (10–99%) and containing 0.01% trifluoroacetic acid (TFA). For the analysis, 20 μL of the sample was injected into the column at 50 °C and a 1.5 mL/min flow rate. The retention time for PTX was 7.0 min. The calibration curve and chromatogram for free PTX are presented in Figure 4A,B, respectively.

**Figure 5:**
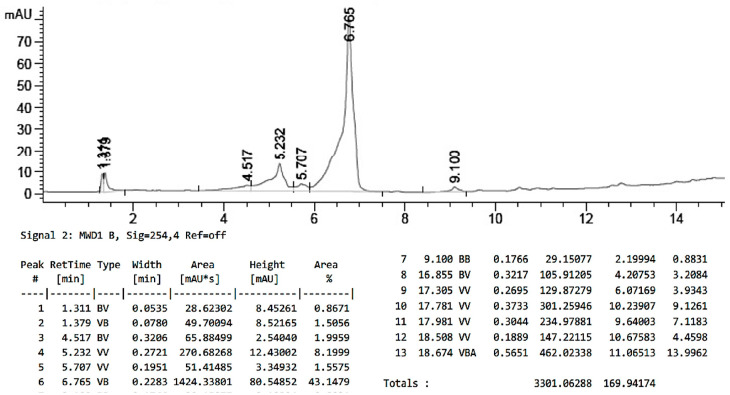
HPLC chromatogram of the hydrolyzed PLGA-PTX NP sample: The peak at 6.765 min represents the major PTX peak.

HPLC analysis of PTX concentration in PLGA-PTX NPs. Shortly, the NP sample was added to 1 mL of acetonitrile and 6 mL of 1 M NaOH and hydrolyzed in a microwave for 15 min at 45 °C. The mixture was then neutralized with 10% HCl and dried using a rotary evaporator. Next, the sample was dissolved in acetonitrile, and 20 μL of the sample was injected into HPLC and analyzed for PTX concentration. The concentration of PTX was determined to be 101.6 µg/mL (3.64 wt.%, N = 3). The chromatogram for free PLGA-PTX NPs is presented in Figure 5.

### 2.10. Stability of PLGA-PTX NPs

The PLGA-PTX NP solution (1.2 mg/mL) in nanopure water or in 10% FBS/nanopure water was incubated at 37 °C. The hydrodynamic diameter of the sample was measured after 0, 2, 4, 6, 24, 48, and 72 h by DLS. The average hydrodynamic diameter of the NPs and polydispersity were established from three independent measurements.

### 2.11. Drug Release Studies

The in vitro PTX release studies from PLGA-PTX NPs were carried out using a dialysis bag diffusion method. Shortly, 168 µL (2.5 mg PLGA in NPs) of PLGA-PTX NPs was dispensed into mini dialysis cups (Slide-A-Lyser MINI dialysis units, Thermo Scientific), and the cups were placed in a beaker containing 600 mL of pH 7.4 phosphate buffer. The samples were gently stirred, and the temperature of the buffer was maintained at 37 ± 1 °C throughout the experiment. Samples (three cups) were withdrawn at defined time points and analyzed for PTX concentration with HPLC. The drug release was calculated from D_t_/D_0_ × 100, where D_t_ and D_0_ indicate the amount of drug released from the NPs at certain intervals and the total amount of drug in the NP solution, respectively.

### 2.12. In Vitro Cell Culture Models

In these studies, both commercially available and patient-derived cell lines (PDCLs) were used. Commercial cell lines A2780, CP70, SKOV-3, OV-90, TOV-21G, ES-2, and OVCAR-3 were purchased from ATCC. The cell lines were cultured in Dulbecco’s Modified Eagle Medium (Sigma Aldrich, St. Louis, MO, USA) supplemented with 10% (A2780, CP70, SKOV-3, ES-2, OVCAR-3) or with 15% (TOV-21G, OV-90) fetal bovine serum (FBS, HyClone) with L-Glutamine (HyClone) and penicillin/streptomycin (HyClone). All cells were grown in a 5% CO_2_, water-saturated atmosphere at 37 °C. For in vitro incubation experiments, 3×10^5^ cells were seeded in each well of a 96-well plate and pre-cultured overnight. The PLGA-PTX NP solution was diluted in growth medium. Stock solution for PTX was prepared in DMSO and diluted in the growth medium to obtain a final concentration of DMSO lower than 0.02%. The incubation time with the cells was 48 h. The cell viability/cytotoxicity was evaluated using a TACS MTT Cell Proliferation assay (Trevigen) according to the manufacturer’s instructions and analyzed using the plate reader (SpectraMax M3 by Molecular devices). The IC_50_ for PLGA-PTX NPs for all cells was established using a dose-response method.

Three PDCLs were derived from independent primary tumors of three patients with high-grade serous ovarian cancer (DPT-89: grade 3, stage IIA; DPT-156: grade 3, stage III and PT217, grade 3, stage IV). Tumor tissues were dissociated to form single-cell suspensions using GentleMACS Octo Dissociator (Miltenyi Biotec, Somerville, MA, USA) following the manufacturer’s protocol. Briefly, tumor tissues were minced with sterile disposable scalpels until the consistency of a smooth paste was obtained. Tissues were re-suspended in GentleMACS C Tubes in 4.7 mL of DMEM medium containing an enzymes mix provided by the manufacturer (Miltenyi Biotec, Somerville, MA, USA). Tubes were placed in the dissociator and processed for 1 h at 37 °C using Program h_tumor_01 designed by the manufacturer. Tubes were then briefly centrifuged at 400× *g* at RT. Dissociated tumors cells were resuspended in 36 mL of DMEM and applied on the MACS SmartStrainer (40 µm). The cell suspension was then centrifuged at 500× *g* for 10 min at RT to precipitate the cell pellet, and two independent cell lines were derived. The resulting cell pellet was then resuspended in growth-conditioned media (CM) in a T25 flask (Greiner Bio-One GmbH, Frickenhausen, Germany). CM were prepared according to a previously published protocol [40]. Briefly, 10 × 10^6^ irradiated Swiss 3T3 J2 mouse fibroblast cells were incubated in cell culture T175 flasks (Greiner Bio-One GmbH, Frickenhausen, Germany) in 30 mL of F medium consisting of gentamicin 10 mg/L, EGF recombinant human protein 10 ug/L, 25% Ham’s F-12 media, 67.5% DMEM, 7.5% Fetal Bovine Serum, 0.5% Penicilin/Streptomycin (Thermo Fisher Scientific, Waltham, MA, USA), Insulin 5.0 mg/L, hydrocortisone 50 µg/L, fungizone 250 µg/L, and cholera toxin 8.4 µg/L (Sigma-Aldrich, St. Louis, MO, USA) for 72 h at 37 °C, 95% humidity and 5% CO_2_ until the cells became confluent. After 72 h, the medium was transferred to 50 mL conical tubes and centrifuged at 300× *g* for 5 min at 4 °C. The supernatant was collected and filtered using a 0.22 mm vacuum filtration system (Thermo Fisher Scientific, Waltham, MA, USA). One part of the filtrated supernatant was mixed with three equal parts of fresh F medium and supplemented with 10 µM of ROCK inhibitor Y-27632 (Selleckchem, Houston, TX, USA). Tumor cells were incubated at 37 °C, 95% humidity, and 5% CO_2_ with the medium replaced every two days. The cells were passaged to near confluence. Cell lines were routinely evaluated for possible mycoplasma contamination by DAPI immunostaining. Cell viability was investigated using an in vitro Toxicology Kit, MTT-based assay (Sigma-Aldrich, St. Louis, MO, USA). The PDCL cells were harvested, and 1000 cells/well were plated in a 96-well plate in 100 µL of CM. The stock solution for PTX was prepared in DMSO and diluted in the growth medium to obtain a final concentration of DMSO lower than 0.02%. The incubation time with the cells was 48 h. The MTT assay was performed three times, and all measurements were performed in triplicate.

### 2.13. Confocal Microscopy Imaging

First, A2780 cells were plated in 8-chamber confocal slides (Nunc Lab Tek Chamber Slides 154534) at a density of 8000 cells per chamber. Next, the cells were incubated for 48 h with PLGA-PTX NPs at 100 nM PTX concentration. After that, the cells were washed 3 times with PBS and fixed with 4% PFA for 7 min at RT. Next, the cells were washed with PBS and 0.01% Triton ×-100 solution for 3 min at RT. After that, the cells were treated with 10% BSA blocking solution for 30 min and washed 3 times with PBS (5 min each). Next, the cells were incubated with 4 µg/mL ***α***-Tubulin-AF488 (Santa Cruz SC-5286 AF488) for 90 min at RT. Then, the cells were washed 3 times with PBS, mounted with mounting medium containing DAPI, and imaged with an LSM 880 Airyscan Fast Live Cell confocal microscope.

## 3. Results and Discussion

### 3.1. Synthesis of PLGA-PTX Hybrid

The PLGA polymer is one of the most readily used core materials to formulate NP for sustained drug delivery applications, especially for cancer therapy [41,42]. Once water enters the PLGA, it triggers hydrolysis of the ester bonds between the lactic acid and glycolic acid units in the polymer. As the ester bonds break, the polymer chains become shorter, eventually resulting in the release of lactic acid and glycolic acid monomers [43]. This degradation is typically slow and controlled, depending on factors like the ratio of lactic to glycolic acid, molecular weight, and environmental conditions [44]. The PLGA breakdown products, lactic and glycolic acid, are non-toxic and can be further metabolized by the body [45]. Importantly, PLGA contains free carboxyl and hydroxyl groups that can be further used to form an ester bond with drug molecules, thus forming PLGA-drug hybrids [46]. The ester bond is labile and is sensitive to conditions such as an acidic or basic environment or enzymatic hydrolysis [47]. In the case of solid tumors, the increased glycolysis combined with low blood flow and increased CO_2_ leads to accumulation of acids and lowering the pH below normal physiological values and results in tumor-surrounding pH as low as 5.5 [48]. This phenomenon combined with the acid-lability of the PLGA ester bonds is beneficial for PLGA NP-mediated drug delivery to solid tumors, as slower PLGA hydrolysis and higher NP stability are expected in circulation, along with accelerated drug release upon fast PLGA hydrolysis once the NPs accumulate in the tumor’s microenvironment.

**Figure 6:**
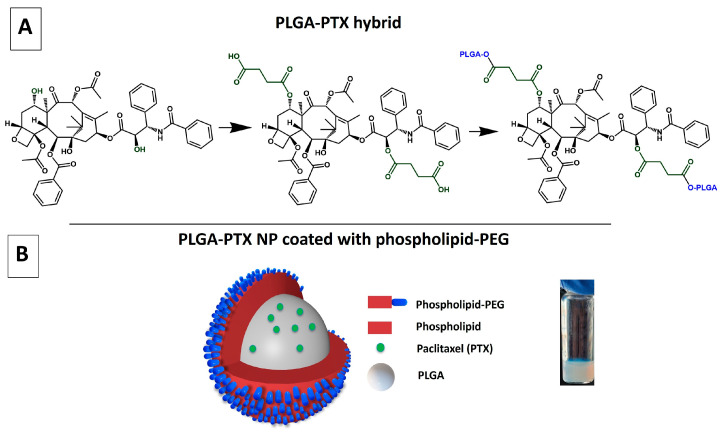
(**A**) Formation of the PLGA-PTX hybrid: modification of PTX with succinic anhydride at the C-2′ and C-7 positions (Step 1), followed by attachment of PLGA via an ester bond to PTX (Step 2). (**B**) Schematic representation of PLGA-PTX nanoparticle (NP) coated with phospholipid-PEG.

To take advantage of the ester bond chemistry, we formed a PLGA-PTX hybrid by esterification reaction between PLGA and PTX. PTX has three hydroxyl groups, however, the reactivity of the hydroxyl groups in PTX can vary depending on their position within the molecule’s structure. Overall, the C-2′ hydroxyl group is generally more reactive due to its accessibility and position, while other hydroxyl groups, like the C-7 hydroxyl, may be less reactive due to steric hindrance and the structure of PTX [49]. Therefore, we introduced a succinic anhydride linker, a short four-carbon cyclic acid anhydride of succinic acid, on PTX to form easily accessible -COOH groups. These were further reacted with hydroxyl groups on PLGA to form labile ester bonds. The strategy is schematically represented in Figure 6. The ratio of PLGA:PTX in the PLGA-PTX hybrid was calculated based on NMR and was found to be 2.2:1, representing high PTX content with respect to PLGA monomers in the hybrid. Interestingly, when the succinic anhydride modification was not used, the PLGA:PTX ratio was significantly lower and was 33:1, indicating less favorable PTX concentration in the PLGA-PTX hybrid. Therefore, the hybrid containing the succinic linker was used for further studies.

### 3.2. Synthesis and Characterization of Physicochemical Properties of PLGA-PTX NPs

**Figure 7:**
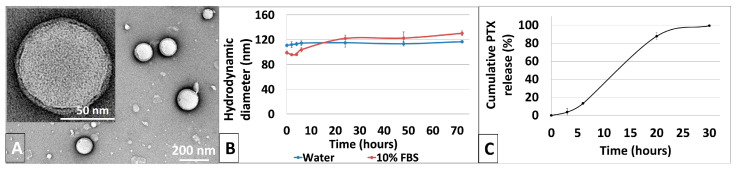
(**A**) TEM image of negatively stained PLGA-PTX NPs. (**B**) Stability of PLGA-PTX NPs in water (blue) and 10% FBS (red). (**C**) In vitro PTX release (%) from PLGA-PTX NPs in PBS at pH 7.4 and 37 °C. Data are presented as mean ± standard deviation (N = 3).

The PLGA-PTX NPs were formed using a nanoprecipitation method [39]. Briefly, a PLGA-PTX hybrid was dissolved in organic solvent and dripped into a hot solution of phospholipid and phospholipid-polyethylene glycol (PEG). The NPs, shown schematically in Figure 6B, formed via a self-assembly process while the solution stirred overnight. Figure 7A shows negatively stained PLGA-PTX NPs. The NPs are spherical in shape and the core diameter, calculated based on TEM images, was found to be 71.7 ± 2.1 nm. The hydrodynamic diameter of the NP, encompassing the core of the particle and a solvation layer, was larger and was determined to be 110.7 nm with low polydispersity of 0.173, as measured by DLS, suggesting uniform size distribution of the NPs. The zeta potential of the NPs was −36.81 ± 2.55 mV. The magnitude of the zeta potential suggests good colloidal stability of PLGA-PTX NPs, as formulations with zeta potentials above 30 mV exhibit stable non-aggregating dispersions. Moreover, it is well established that the PEG coating on the NP can reduce protein binding in the blood and help avoid recognition of the NPs by the reticular endothelial system (RES), contributing to efficient NP-mediated drug delivery to the cells [50,51].

To further examine the long-term stability of PLGA-PTX NPs, they were suspended in water and in 10% fetal bovine serum solution in water. The diameter and polydispersity index were monitored by DLS. Over a span of 72 h, the hydrodynamic diameter of the NPs did not significantly change in either water or FBS solution, as shown in Figure 7B. The polydispersity of the NPs (Figure S2) remained relatively constant, not exceeding 0.25, confirming good stability of the NP formulation over time. This is consistent with previous reports where NPs with high negative surface charges presented the highest kinetic stability [52], suggesting resistance to aggregation, which is a critical parameter in biological applications of NP systems.

The PTX loading in the PLGA-PTX NPs as well as PTX release over time were measured using HPLC. The initial PTX concentration in the NPs was established to be 3.64 wt.%. PLGA undergoes hydrolytic degradation when exposed to water, which is expected to liberate PTX upon hydrolysis of PLGA-PTX ester linkage. The PTX release was monitored over 24 h, and the results are shown in Figure 7C. Shortly, the PLGA-PTX solution was placed in dialysis cassettes and suspended in PBS buffer with gentle stirring at 37 °C. After 3 h, only 5% of PTX was released from the NPs, which increased to just 12% after a total of 6 h. The NPs provided a sustained release of PTX with 88% release observed after 20 h. This result suggests that the PLGA-PTX NPs are a slow-releasing system without burst release, which was expected for the PLGA polymer due to its unique degradation characteristics. PLGA is composed of lactic acid (PLA) and glycolic acid (PGA) monomers. The polymer slowly breaks down into PLA and PGA monomers through hydrolysis, allowing a controlled release of encapsulated drugs that slowly diffuse through the polymer matrix as it swells and degrades [53]. This diffusion is gradual, allowing steady drug release, as demonstrated with PTX. This is highly beneficial in the context of drug delivery systems, where the NPs could provide a sustained therapeutic effect, reducing the need for frequent administration. In addition, the degradation rate of PLGA-based NPs can be controlled by a ratio of lactic to glycolic acid in the polymer, and the higher ratio results in slower polymer degradation [43]. The more hydrophobic nature of lactic acid-rich PLGA limits the water penetration into the matrix, which slows down the hydrolytic cleavage of the ester bonds [43]. Thus, the release of drugs encapsulated within PLGA via ester bonds, such as PTX, could be further modulated.

### 3.3. In Vitro Evaluation of PLGA-PTX NPs

**Figure 8:**
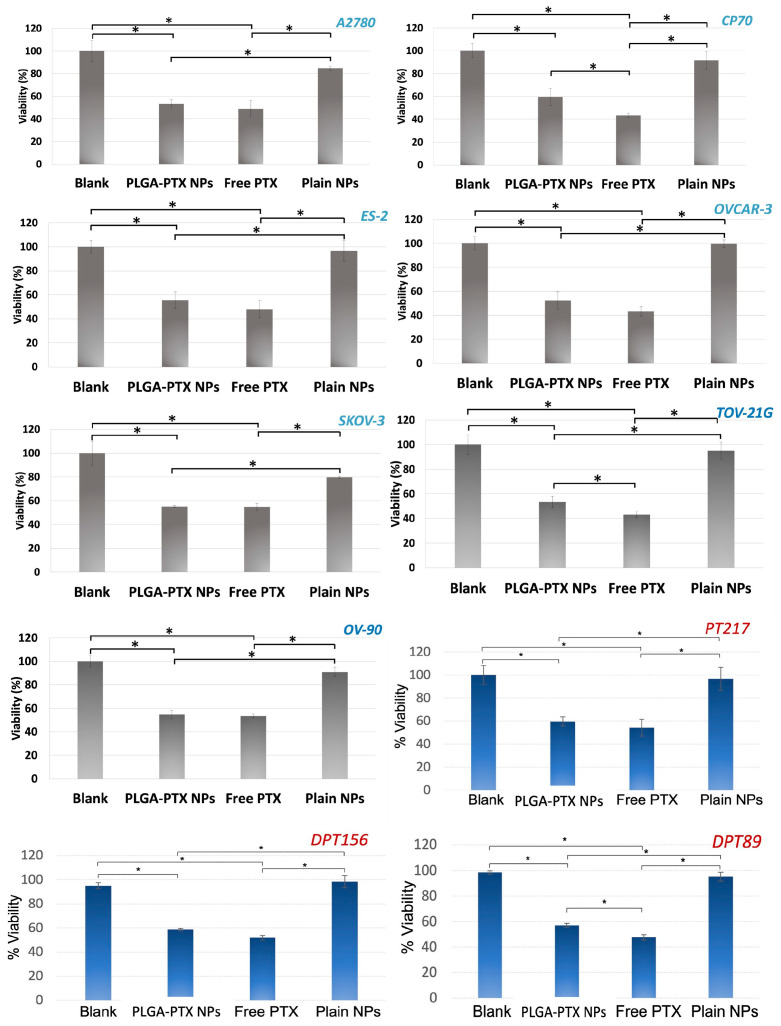
In vitro viability with PTX-PLGA NP. Each column represents the mean and standard deviation of N = 3 with *p* < 0.005 (* in SKOV-3, OVCAR-3, OV-90, TOV-21G, ES-2, A2780, DPT156, PT217), and *p* < 0.03 (* in CP70, DPT89). The concentrations correspond to IC50 values of PTX for each cell line and are as follows: 100 nM (A2780), 75 nM (TOV-21G, SKOV-3, and CP70), 125 nM (ES-2), 50 nM (OVCAR-3), 700 nM (OV-90), 150 nM (PT217, DPT89, and DPT156).

Next, we evaluated the biological activity of the PLGA-PTX NPs in vitro using a panel of OvCA cell lines with different histological subtypes and genetic background to reflect the heterogeneity of OvCA (Figure 8). Also, we included patient-derived OvCA cells in this study that more closely resemble the actual OvCA cells, which allows for more accurate testing of drug efficacy, chemoresistance, and therapeutic response. Notably, all PDCLs were derived from women with high-grade serous OvCA at different stages (Materials and Methods), and in particular, PT217 was from a platinum-resistant patient. In this study, we incubated the cells with PLGA-PTX NPs for 48 h before testing their viability using an MTT assay. We used plain NPs (without PTX), free PTX, and cells only as controls. In all OvCA cells tested, the PLGA-PTX NPs were cytotoxic to the cells, resulting in lowering the cellular viability to ~50%, which was comparable to free drug results. The plain NPs and PBS did not decrease the viability of the cells. Importantly, while the PLGA-PTX NPs were stable in aqueous solution, offering significant advantage for biological applications over a free PTX, which was added as a suspension. Collectively, these data demonstrate that the PLGA-PTX NPs’ formulation is efficacious towards OvCA cells, even those PDCLs derived from women with late-stage tumors, which are generally chemoresistant.

**Figure 9:**
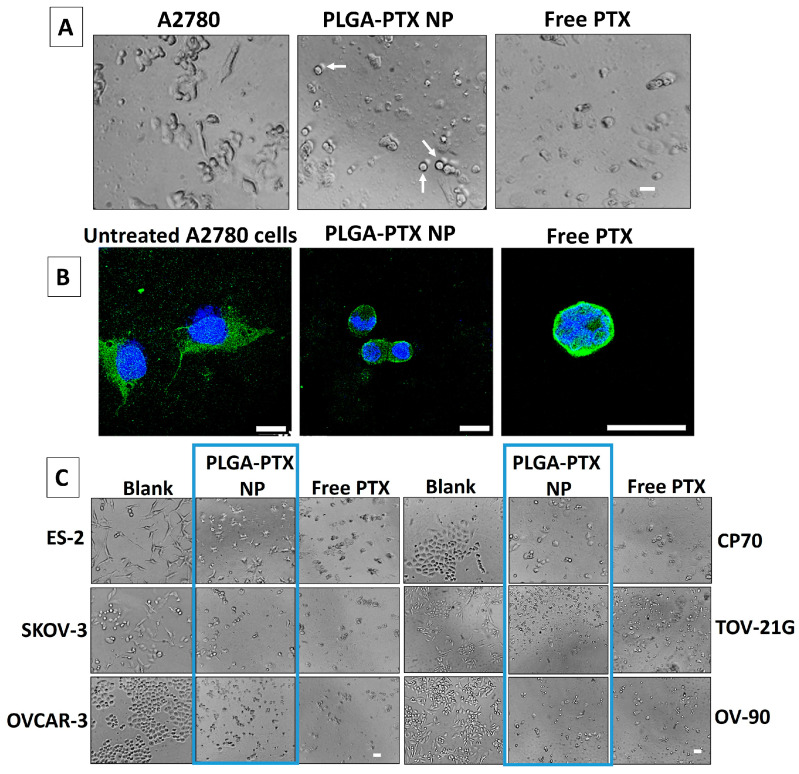
(**A**) Optical microscopy images of the A2780 ovarian cancer cell line treated with free PTX or PLGA-PTX NPs (blue boxes), both at the same concentration of 100 nM. Images were taken after 48 h of incubation. Arrows indicate changes in cell shape. The scale bar represents 40 µm. (**B**) Laser-scattering confocal microscopy images of the A2780 ovarian cancer cell line incubated with PLGA-PTX NPs or the free drug, both at the same concentration of 100 nM. Nuclei were stained with DAPI (blue), and tubulin was stained with α-Tubulin-AF488 (green). A merged image of all channels is shown. All scale bars represent 20 µm. (**C**) Optical microscopy images of six ovarian cancer cell lines treated with free PTX or PLGA-PTX NPs at the following concentrations: 100 nM (A2780), 75 nM (TOV-21G, SKOV-3, and CP70), 125 nM (ES-2), 50 nM (OVCAR-3), and 700 nM (OV-90). Images were taken after 48 h of incubation. The scale bar represents 40 µm.

Microtubules play a crucial role not only in various cellular processes but also in determining and maintaining the shape and structure of the cell. As part of the cytoskeleton, microtubules provide structural support and enable cells to maintain their shape, forming a network that supports the cell membrane, preventing the cell from collapsing or deforming and contributing to its overall architecture [54]. Also, microtubules are dynamic structures that constantly undergo polymerization and depolymerization, which is inhibited by PTX that binds to microtubules, stabilizing them by preventing their depolymerization and thus leading to a non-dynamic state [55]. This affects the cell’s ability to change shape or migrate because the cytoskeleton cannot dynamically reorganize to form protrusions, and the cells may appear more rounded or flattened [56]. We evaluated the influence of PLGA-PTX NPs on the shape of the cells to further corroborate the biological activity of PTX while in NP formulation. Shortly, we incubated the cells with PLGA-PTX NPs in the same manner as for in vitro studies, and we compared the cells incubated with PLGA-PTX NPs to cells incubated with free PTX as well as untreated cells. The results are presented in Figure 9A,C. The untreated cells, such as A2780, ES-2, SKOV-3, TOV-21G, and OV-90 are oval or more elongated and polygonal, and after incubation with PLGA-PTX NPs as well as with free PTX, the cells lost their shape and became more rounded and spherical, and even shrank. The shape change was also observed for OVCAR-3 and CP-70 cells that before treatment were irregular or rounded, but after incubation with PLGA-PTX NPs and free PTX, the cells became disorganized and even swollen, with some cell aggregates. To further corroborate the shape change, we used confocal microscopy, where representative cell line A2780 was imaged after treatment with PLGA-PTX NPs and PTX only under the same conditions as in the above studies. The microtubules were stained green, and the nuclei were blue, and the images are shown in Figure 9B and Figure S4. It can be clearly seen that the cells lose the structural support provided by the microtubules, which changed from long filaments to shrunken and bundled clusters around the nucleus. These studies indicate that the PLGA-PTX NPs are biologically active and effectively stabilize microtubules, comparably to the free drug.

## 4. Conclusions

In summary, a PLGA-PTX NP was developed to provide an alternative treatment option to overcome the high systemic toxicity which limits the use of free PTX chemotherapy in cancer patients. The NPs were synthesized from a PLGA-PTX hybrid that was formed after chemical modification of the PLGA polymer with linker “activated” PTX that resulted in a high ratio of PTX content in the hybrid. The PLGA-PTX NPs encapsulated PTX within the core using a labile ester bond, and the platform was determined to be a stable and slow-releasing system. The PLGA-PTX NPs were biologically active in ovarian cancer cultures that included patient-derived models established from women with late-stage, high-grade serous ovarian cancer. Of particular interest, one of these was derived from a patient with platinum-resistant disease. Overall, the PLGA-PTX NP formulation provides a new approach to efficient PTX treatment comparable to a free therapeutic with a potential to greatly improve the therapy.

## Data Availability

Data are contained within the article and Supplementary Materials.

## Supplementary Materials

The following supporting information can be downloaded at: https://www.mdpi.com/article/10.3390/pharmaceutics17060689/s1: HPLC analysis of PTX concentration in PLGA-PTX NPs synthesized from PLGA-PTX hybrid without a succinic acid linker; polydispersity of PLGA-PTX NPs with linker in water and 10% FBS; IC50 determination for PLGA-PTX NPs (synthesized from PLGA-PTX hybrid with linker) in 7 ovarian cancer cell lines and 3 patient-derived cell lines; confocal microscopy results with split channels for A2780 and OVCAR-3 after incubation with PLGA-PTX NPs, and Imaris reconstruction for OVCAR-3 cell lines (including images and video).

## Abbreviations

The following abbreviations are used in this manuscript:

NP	Nanoparticle
PTX	Paclitaxel, Taxol
PLGA	Poly(lactic)co-(glycolic)acid
PEG	Polyethylene glycol
DLS	Dynamic Light Scattering
TEM	Transmission Electron Microscopy
FBS	Fetal Bovine Serum
HPLC	High Performance Liquid Chromatography
LC-MS	Ultra High-Performance Liquid Chromatography
^1^H NMR	Proton Nuclear Magnetic Resonance
