# Supplementary material for: Synthesis and Characterization of Poly(Lactic-Co-Glycolic Acid)–Paclitaxel (PLGA-PTX) Nanoparticles Evaluated in Ovarian Cancer Models
Source: Pharmaceutics. 2025 May 23;17(6):689. doi: 10.3390/pharmaceutics17060689 (PMC12196068; doi:10.3390/pharmaceutics17060689)
Supplement: Supplementary file 1 [file pharmaceutics-17-00689-s001.zip › pharmaceutics-3582625-supplementary.pdf]

Synthesis and Characterization of Poly(lactic-co-glycolic acid)–Paclitaxel (PLGA-PTX) Nanoparticles Evaluated in Ovarian Cancer Models

Sylwia A. Dragulska [1], Maxier Acosta Santiago [1], Sabina Swierczek [2,4], Linus Chuang [2], Olga Camacho-Vanegas[3], Sandra Catalina Camacho[3], Maria M. Padron-Rhenals [3], John Martignetti [2,3], and Aneta J. Mieszawska [1,*]

1 Department of Chemistry and Biochemistry, Brooklyn College, Brooklyn, NY 11210, USA
2 Rudy L. Ruggles Biomedical Research Institute, Nuvance Health, Danbury, Connecticut 06810, USA
3 Department of Genetics and Genomic Sciences, Icahn School of Medicine at Mount Sinai, New York, NY 10029, USA
4 Department of Obstetrics, Gynecology and Reproductive Sciences, Larner College of Medicine, University of Vermont, Burlington, VT, USA

* Correspondence: aneta.mieszawska@brooklyn.cuny.edu

HPLC analysis of PTX concentration in PLGA-PTX NPs using the PLGA-PTX hybrid directly conjugated via an ester bond.

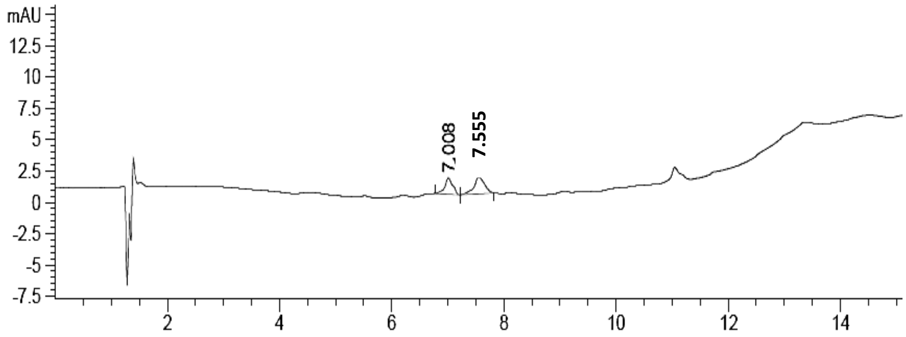

Signal 2: MWD1 B, Sig=254,4 Ref=off

Peak #	RetTime [min]	Type	Width [min]	Area [mAU*s]	Height [mAU]	Area %
1	7.008	MM	0.1621	12.73539	1.30936	41.9578
2	7.555	MM	0.2161	17.61744	1.35896	58.0422
Totals :				30.35284	2.66833	

Figure S1. HPLC chromatogram of hydrolyzed PLGA-PTX NP sample: peaks at 7.008; 7.555 min (PLGA-PTX hybrid without linker) represents major peaks for PTX.

Polydispersity of PLGA-PTX NP with linker.

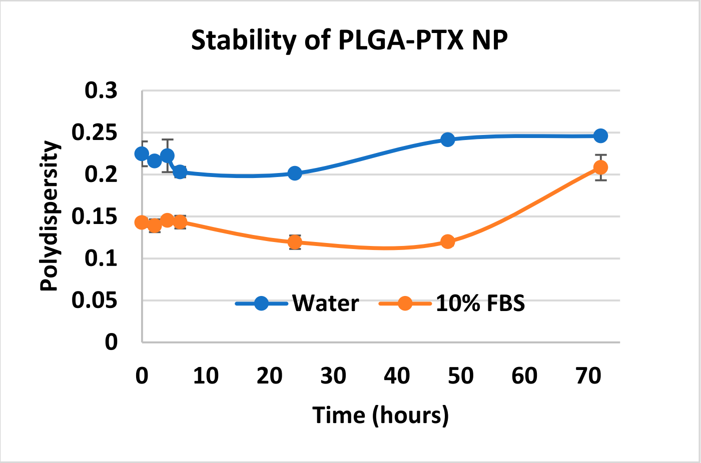

Figure S2. The stability of PLGA-PTX NP with linker was tested in water and 10% FBS over 72 hours.

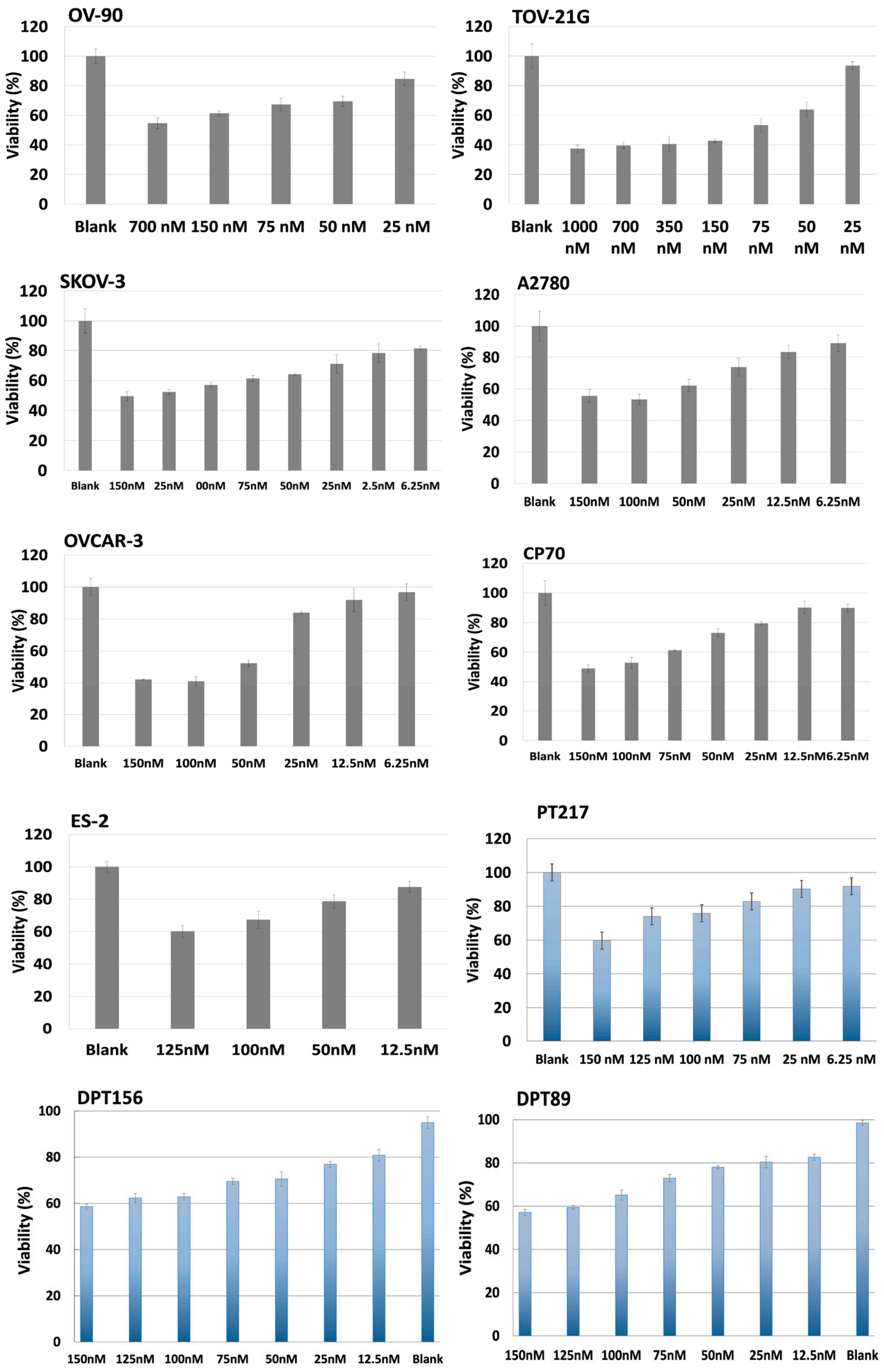

Figure S3 Determination of IC50 of PLGA-PTX NPs using 5-10 kDa PLGA-PTX hybrid conjugated with succinic acid, for all cell lines.

Confocal microscopy

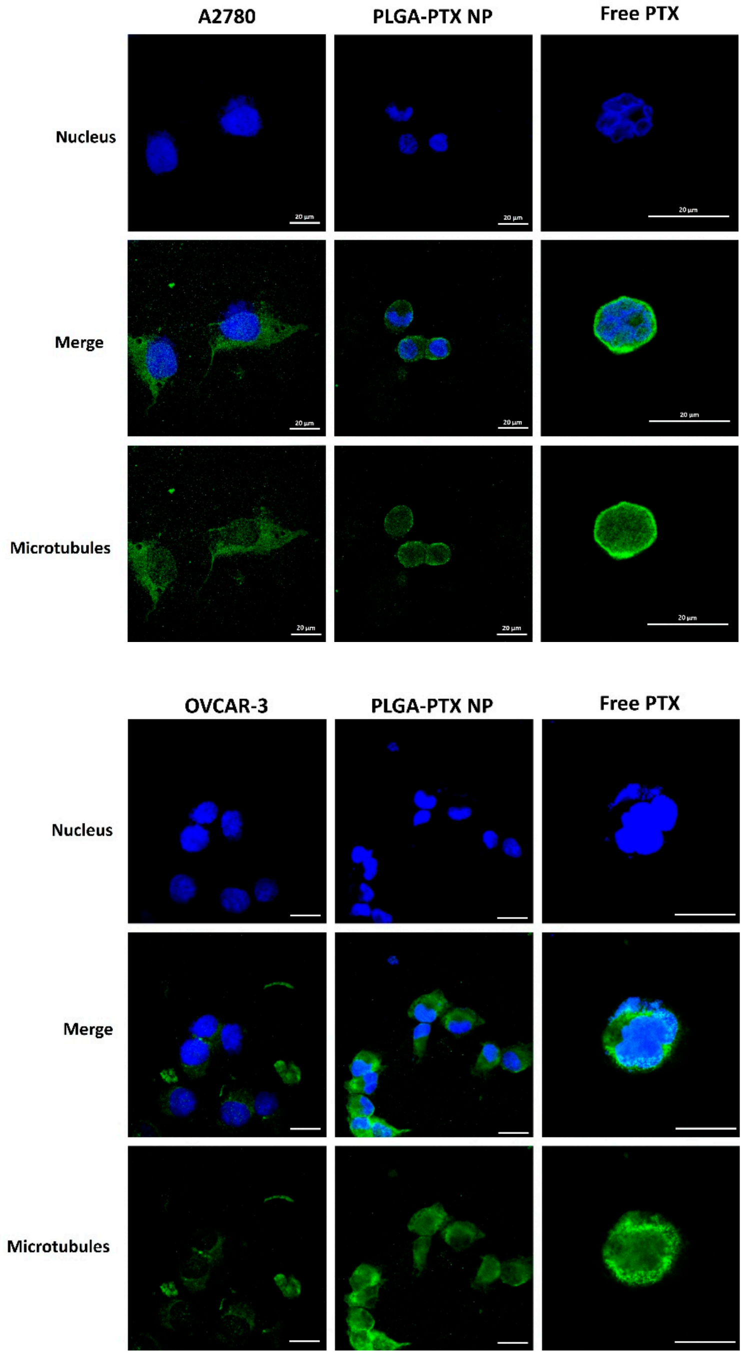

Figure S4. Laser scanning confocal microscopy imaging of A2780 and OVCAR-3 cells incubated with PLGA-PTX NP or free PTX, with stained nuclei (DAPI, blue), tubulin (α-tubulin-AF488, green), and the merged channels. The concentrations of PTX were 100 nM for A2780 and 50 nM for OVCAR-3 cell lines, respectively. All scale bars (white) represent 20 μm.

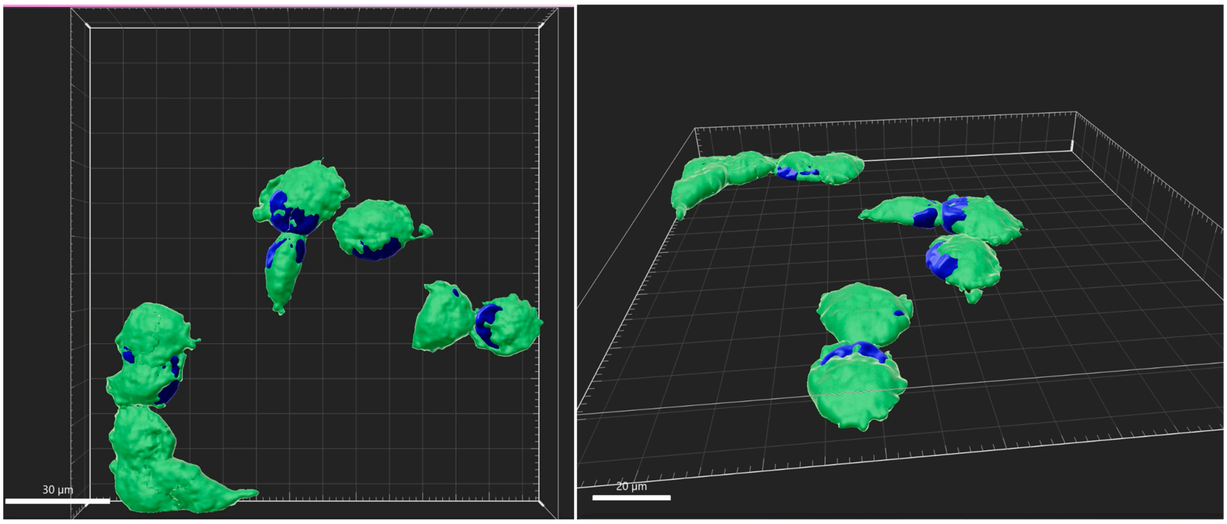

Figure S5. Imaris projection of OVCAR-3 cells based on Z-stack confocal microscopy imaging for cells incubated with PLGA-PTX NP. Nuclei are stained with DAPI (blue), and tubulin is stained with α-tubulin-AF488 (green). The scale bar is 30 µm.
